# On Modern Methods of Treating Fractures

**Published:** 1916-12

**Authors:** 


**On Modern Methods of Treating Fractures.** By Ernest W. Hey Groves, M.S., M.D., B.Sc. Lond. Pp. Bristol : John Wright & Sons Ltd. Price 7s. 6d. net.

This thoroughly original work brings to bear great mechanical ingenuity, experience and skill upon a subject that might have been thought threadbare. It is a record of one of the most important advances that British surgery has made during the war. Both for civil and military practice the author's novel methods are worthy of careful attention. The illustrations are very good, the text clear and concise, and forms a very able presentment of a department of surgery which the author has done much to advance.

